# Ras in epidermal proliferation

**DOI:** 10.18632/oncotarget.2275

**Published:** 2014-07-29

**Authors:** Matthias Drosten, Carmen G. Lechuga, Mariano Barbacid

**Affiliations:** Molecular Oncology Programme, Centro Nacional de Investigaciones Oncológicas (CNIO), Madrid, Spain

The Ras family of small GTPases (H-Ras, N-Ras and K-Ras) constitutes a central node in the transmission of mitogenic signals from cell surface receptors to the cell cycle machinery. In general, activation of membrane receptors causes GTP loading of Ras proteins via guanosine nucleotide exchange factor (GEF) stimulation. Loading of GTP promotes conformational changes that ultimately allow binding and subsequent activation of a battery of effector molecules, such as the Raf kinases, the catalytic subunits of phosphatidylinositol 3-kinase (PI3K) or the Ral-GEF family of proteins. Ras-mediated signaling is terminated upon GTP hydrolysis, a reaction that leads to an inactive conformation characterized by a GDP-bound state. This hydrolysis is catalyzed by the intrinsic GTPase activity of Ras proteins, a rather slow reaction that can be accelerated by a familiy of GTPase-activating proteins (GAPs) [1].

To date, little is known about the physiological functions of wild-type Ras proteins, since the majority of investigations focused on the role of their oncogenic counterparts [2]. In addition, the genetic analysis of the role of homeostatic Ras signaling has been complicated by the high redundancy between H-, N- and K-Ras proteins. To overcome this limitation, we have developed mice as well as cell lines that express conditional *K-Ras* alleles in the absence of *H-Ras* and *N-Ras* loci. Ablation of these conditional *K-Ras* alleles renders cells (and mice) Rasless, that is devoid of all Ras isoforms. Rasless mice die of multiorgan failure in less than two weeks (our unpublished observations). Likewise, Rasless fibroblasts are incapable of proliferating even in the presence of growth factors [3].

**Figure 1:**
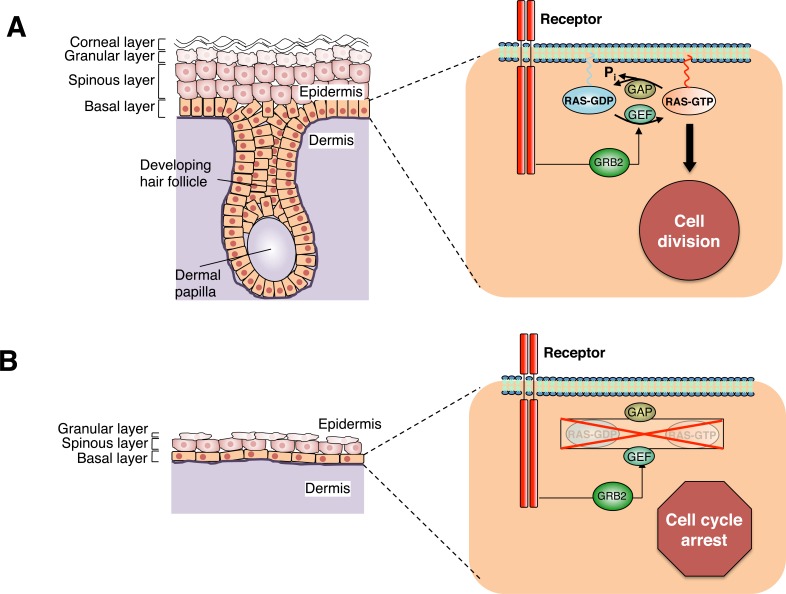
Ras signaling in epidermal keratinocytes (A) Schematic representation of epidermal development at embryonic day E18.5 in wild-type mouse embryos. The developing hair follicle has already surrounded mesenchymal cells that form the dermal papilla. Basal, spinous, granular and corneal layers of epidermis as well as the dermis are indicated. Magnification: Basal layer keratinocyte and Ras-driven signaling events. Activation of cell surface receptors causes GTP-loading of Ras proteins via stimulation of GEFs, which is mediated by adaptor proteins such as GRB2. GTP-loaded Ras activates the Raf/Mek/Erk cascade to drive keratinocyte proliferation. GAPs facilitate GTP hydrolysis and termination of active signaling. (B) Schematic representation of epidermal development at embryonic day E18.5 in H-, N-, and K-Ras triple knock-out mouse embryos. Due to the lack of proliferation of the cells in the basal layer, the epidermis fails to develop properly. Basal, spinous, granular and corneal layers of epidermis as well as the dermis are indicated. Magnification: Basal layer keratinocyte lacking Ras proteins.

In an effort to better understand the role of Ras signaling *in vivo*, we have focused on the skin epidermis, since this tissue is highly proliferative, and there are well-defined markers for cell proliferation as well as for the various stages of cell differentiation. To selectively eliminate all three Ras genes from the epidermis, we crossed our *H-Ras*^−/−^;*N-Ras*^−/−^;*K-Ras*^lox/lox^ mice with a strain expressing a Cre recombinase under the control of the keratin 5 promoter, a gene that becomes activated in the basal layer of the epidermis during early embryonic development [4]. We observed that mice lacking the three Ras isoforms in the epidermis were not viable after birth due to a dramatic thinning of the epidermis during embryonic development (Figure 1).

The epidermis consists of various layers of keratinocytes with varying degree of proliferation and differentiation [5]. Normally, only keratinocytes in the basal layer are actively proliferating, and either maintain the basal layer through symmetric cell divisions or initiate the process of stratification through asymmetric divisions. Embryos lacking the three Ras isoforms in the epidermis display an almost complete cell cycle arrest in the basal layer, thus indicating a fundamental role of Ras signaling in these cells. Interestingly, we detected increased expression of the Cyclin-dependent kinase inhibitors p15^INK4b^ and p21^Cip1^ in all cells of the arrested basal layer. This was accompanied by downregulation of two transcription factors usually expressed at high levels in the basal layer: c-Myc and ΔNp63. Further mechanistic characterization of cultured keratinocytes revealed that this regulation occurred largely at the transcriptional level.

Surprisingly, we did not observe increased differentiation of the epidermal keratinocytes lacking Ras proteins, thus indicating that Ras signaling, although crucial for keratinocyte proliferation, does not directly control the differentiation state of keratinocytes *in vivo* (4). In fact, our data indicate a delay in stratification, probably owing to the lack of proliferation in the basal layer. In addition, these observations also indicate that proliferation and differentiation are not directly connected, since fully arrested keratinocytes in the basal layer do not differentiate prematurely (4). Not surprisingly, we also detected a striking delay in hair follicle development. This process initially requires a burst of proliferation, especially in the downgrowing follicle, thus indicating that the lack of Ras signaling dramatically interferes with the tight regulation of follicle development [4].

A number of tyrosine kinase receptors have previously been linked to epidermal homeostasis. Indeed, genetic evidence indicates that the epidermal growth factor receptor (EGFR) plays a crucial role in epidermal maintenance. Mice lacking the EGFR display a substantially thinner epidermis than control mice as well as defects in keratinization and hair follicle development [6]. However, ablation of the EGFR does not completely block proliferation of epidermal keratinocytes as seen in mice lacking the three Ras isoforms. These observations suggest that signaling via EGFR and possibly through other receptors such as the fibroblast growth factor receptor or the insulin/insulin-like growth factor receptors may converge upon Ras proteins.

Ras proteins are known to activate multiple effector pathways [1]. Yet, mitogenic signaling is suspected to be mediated by the MAP kinase cascade, which involves sequential activation of three families of serine/threonine kinases, Raf, Mek and Erk. Interestingly, ablation of the Mek kinases from the epidermis provided a similar phenotype to that observed in mice lacking the three *Ras* isoforms [7]. Moreover, ectopic expression of a constitutively active Erk kinase, the last component of the MAP kinase cascade, rescued the proliferation defect of keratinocytes lacking Ras, at least *in vitro* [4]. These genetic data, taken together, strongly support the concept that the Raf/Mek/Erk pathway is a critical downstream element of Ras signaling in epidermal homeostasis (Figure 1).
